# Supplementary material for: From Field Tests to Molecular Tools—Evaluating Diagnostic Tests to Improve Rabies Surveillance in Namibia
Source: Viruses. 2023 Jan 28;15(2):371. doi: 10.3390/v15020371 (PMC9966532; doi:10.3390/v15020371)
Supplement: Supplementary file 1 [file viruses-15-00371-s001.zip › viruses-2185909-supplementary.pdf]

Supplementary Table S1. Details of Namibian samples analyzed in this study.

				Test results			
				FAT		qPCR FLI	
**FLI-ID N°**	**Species**	**year**	**CVL-ID N°**	CVL compiled	**FLI compiled**	**ct value**	**result**
46852	Feline Wild	2019	2727	POS	POS	22.33	POS
46853	Jackal	2019	2169	POS	POS	17.70	POS
46854	Donkey	2019	1782	POS	POS	21.13	POS
46855	Black Rhino	2018	563	NEG	POS	36.32	POS
46856	Dog	2019	563	NEG	NEG	40.17*	NEG
46857	Meerket	2018	3671	NEG	NEG	N/A	NEG
46858	White Rhino	2018	4863	POS	POS	17.32	POS
46859	Dog	2018	3499	POS	POS	21.74	POS
46860	Dog	2019	561	NEG	NEG	N/A	NEG
46863	Jackal	2019	2045	NEG	NEG	N/A	NEG
46864	Jackal	2019	2042	POS	POS	21.87	POS
46865	Jackal	2019	1307	POS	POS	21.40	POS
46866	Jackal	2019	2388	POS	POS	19.28	POS
46867	Ovine	2018	897	NEG	NEG	N/A	NEG
46868	Ovine	2018	4181	NEG	NEG	36.66*	NEG
46869	Jackal	2018	3116	POS	POS	16.36	POS
47122	Eland	2018	5737	NEG	NEG	N/A	NEG
47123	Ovine	2019	1871	NEG	NEG	N/A	NEG
47124	Ovine	2019	1218	POS	POS	21.00	POS
47125	Eland	2019	2242	POS	POS	20.25	POS
47126	Hyena	2018	4000	NEG	NEG	39.42*	NEG
47127	Lion	2019	2389	NEG	NEG	31.28	POS
47128	Eland	2018	2353	NEG	NEG	N/A	NEG
47129	Sable Antilope	2019	968	NEG	NEG	N/A	NEG
47130	Ovine	2019	1280	POS	POS	20.65	POS
47131	Ovine	2018	322	NEG	NEG	N/A	NEG
47132	Porcine	2018	1695	NEG	NEG	16.01	POS
47133	Hyena	2018	2201	NEG	NEG	40.44*	NEG
47134	Oryx	2018	100	NEG	NEG	N/A	NEG
47135	Nyala	2019	2963	NEG	NEG	N/A	NEG
47136	Jackal	2018	2599	POS	POS	21.26	POS
47138	Equine	2019	560	NEG	NEG	N/A	NEG
47139	Porcine	2019	941	NEG	NEG	N/A	NEG
47140	Porcine	2018	3851	NEG	NEG	N/A	NEG
47141	Zebra	2018	4502	NEG	NEG	36.18*	NEG
47142	Eland	2018	01404	POS	POS	20.01	POS
47167	Bovine	2019	1516	POS	POS	19.85	POS
47168		2019	753	NEG	NEG	N/A	NEG

47169	Kudu	2018	2476	POS	POS	26.18	POS
47170	Bovine	2019	861	NEG	NEG	N/A	NEG
47171	Bovine	2019	2240	NEG	NEG	N/A	NEG
47172	Kudu	2018	4793	POS	POS	19.27	POS
47173		2018	4841	n.d.	NEG	17.79	POS
47174	Bovine	2019	400	NEG	NEG	N/A	NEG
47175	Kudu	2019	1292	POS	POS	18.35	POS
47176	Bovine	2019	2174	NEG	NEG	41.00*	NEG
47177	Kudu	2019	577	POS	POS	20.91	POS
47178	Bovine	2019	2901	POS	POS	18.31	POS
47179	Bovine	2018	4055	POS	POS	19.23	POS
47180	Bovine	2019	491	POS	NEG	N/A	NEG
47181		2019	2174	NEG	NEG	23.51	POS
47182	Bovine	2019	2391	POS	POS	18.27	POS
47183	Kudu	2018	3813	POS	POS	28.20	POS
47184	Bovine	2019	4803	POS	POS	20.04	POS
47185	Bovine	2019	483	NEG	POS	22.34	POS
47186	Kudu	2018	3595	POS	POS	20.11	POS
47187	Bovine	2019	2568	NEG	NEG	34.46	POS
47188	Bovine	2019	2999	POS	POS	17.84	POS
47190	Bovine	-	836	POS	POS	19.99	POS
47191	Kudu	2018	151	POS	POS	18.73	POS
47192	Bovine	2018	6328	POS	POS	18.21	POS
47193	Kudu	2018	3286	POS	POS	20.29	POS
47194	Kudu	2018	100	POS	POS	19.85	POS
47195	Kudu	2018	2600	NEG	NEG	35.05*	NEG
47196	Kudu	2019	2090	POS	POS	20.19	POS
47197	Kudu	2019	399	POS	POS	17.48	POS
47198	Kudu	2019	528	POS	POS	19.75	POS
47199	Bovine	2019	1101	NEG	NEG	39.18*	NEG
47200	Bovine	-	831	NEG	NEG	37.52*	NEG
47201	Bovine	2019	1769	POS	POS	20.57	POS
47202	Bovine	2019	2241	POS	POS	19.53	POS
47203	Bovine	-	625	POS	POS	20.98	POS
47204	Bovine	2019	2243	POS	POS	18.11	POS
47205	Kudu	2018	3613	POS	POS	19.13	POS
47206	Bovine	2018	4612	POS	POS	19.56	POS
47207	?	2019	791	NEG	NEG	36.48*	NEG
47208	Bovine	2019	1329	POS	POS	20.07	POS
47209	Bovine	-	2933	POS	POS	16.73	POS
47210	Bovine	2019	2856	POS	POS	18.08	POS
47211	Kudu	2019	2684	POS	POS	20.18	POS
47212	Kudu	2018	3351	POS	POS	20.18	POS
47213	Kudu	2018	2321	POS	POS	18.78	POS
47214	Bovine	2019	2092	NEG	NEG	33.66	POS
47215	Bovine	2019	23	POS	POS	19.20	POS
47216	Kudu	2019	2417	POS	POS	20.70	POS

47217	Kudu	2019	226	POS	POS	20.92	POS
47218	Bovine	2019	3000	NEG	NEG	N/A	NEG
47219	Bovine	2018	4386	POS	POS	15.33	POS
47220	Kudu	2019	1188	POS	POS	15.35	POS
47221	Bovine	2019	2628	NEG	NEG	N/A	NEG
47222	Bovine	2019	2416	POS	POS	19.21	POS
47223	Bovine	2019	1404	POS	POS	17.65	POS
47224	Kudu	2018	4679	POS	POS	20.41	POS
47225	Bovine	2019	665	NEG	NEG	33.20	POS
47226	Bovine	2019	830	NEG	NEG	N/A	NEG
47251	Kudu	2018	6579	POS	POS	15.69	POS
47252	Kudu	2019	1761	POS	POS	18.12	POS
47253	Bovine	2019	1641	POS	POS	19.24	POS
47254	Bovine	2019	3030	NEG	NEG	N/A	NEG
47255	Kudu	2019	593	POS	POS	17.52	POS
47256	Kudu	2019	285	POS	POS	13.43	POS
47257	Kudu	2018	2551	POS	POS	19.15	POS
47258	Kudu	2018	3351 2x	POS	POS	15.84	POS
47259	Bovine	2019	2171	NEG	NEG	N/A	NEG
47260	Bovine	2019	58	POS	POS	16.67	POS
47261	Canine	2019	1365	NEG	NEG	N/A	NEG
47262	Canine	2019	1672	NEG	NEG	N/A	NEG
47263	Caprine	2019	2936	NEG	NEG	N/A	NEG
47264	Caprine	2019	482	NEG	NEG	N/A	NEG
47265	Kudu	2019	49	NEG	NEG	N/A	NEG
47266	Canine	2019	1540	NEG	NEG	N/A	NEG
47267	Kudu	2019	249	NEG	NEG	N/A	NEG
47268	Canine	2019	2043	NEG	NEG	N/A	NEG
47269	Caprine	2018	4135	NEG	NEG	N/A	NEG
47270	Caprine	-	4222	NEG	NEG	N/A	NEG
47271	Caprine	2019	1783	NEG	NEG	N/A	NEG
47272	Kudu	2019	2741	NEG	NEG	N/A	NEG
47273	Canine	2019	1689	NEG	NEG	N/A	NEG
47274	Canine	2019	693	NEG	NEG	N/A	NEG
47275	Caprine	2019	2457	NEG	NEG	N/A	NEG
47276	Canine	2019	833	NEG	NEG	N/A	NEG
47277	Canine	2019	2371	NEG	NEG	N/A	NEG
47278	Canine	2019	1875	NEG	NEG	N/A	NEG
47279	Canine	2019	2857	NEG	NEG	N/A	NEG
47280	Canine	2019	2855	NEG	NEG	N/A	NEG
47281	Canine	2019	2044	NEG	NEG	N/A	NEG
47282	Canine	2019	692	NEG	NEG	N/A	NEG
47283	Canine	2019	2900	NEG	NEG	N/A	NEG
47284	Caprine	2018	6510	NEG	NEG	N/A	NEG
47285	Kudu	2019	1517	NEG	NEG	N/A	NEG
47286	Canine	2019	569	NEG	NEG	N/A	NEG
47287	Caprine	2018	3988	NEG	NEG	N/A	NEG

47288	Canine	2019	2238	NEG	NEG	N/A	NEG
47289	Bovine	2019	1872	NEG	NEG	N/A	NEG
47290	Canine	2019	2060	NEG	NEG	29.69	POS
47291	Canine	2019	1620	NEG	NEG	N/A	NEG
47292	Canine	2019	832	NEG	NEG	N/A	NEG
47293	Caprine	2019	75	NEG	NEG	19.29	POS
47294	Canine	2019	1691	NEG	NEG	N/A	NEG
47295	Canine	2019	2555	NEG	NEG	N/A	NEG
47296	Canine	2019	2553	NEG	NEG	N/A	NEG
47297	Caprine	2019	2554	NEG	NEG	N/A	NEG
47298	Canine	2019	694	NEG	NEG	N/A	NEG
47299	Caprine	2019	82	NEG	NEG	N/A	NEG
47301	Canine	2019	2485	NEG	NEG	N/A	NEG
47302	Canine	2019	1366	NEG	NEG	N/A	NEG
47303	Canine	2019	1902	NEG	NEG	N/A	NEG
47304	Canine	2019	737	NEG	NEG	N/A	NEG
47305	Canine	2019	710	NEG	NEG	N/A	NEG
47306	Canine	2019	1760	NEG	NEG	N/A	NEG
47307	Canine	2019	2764	NEG	NEG	N/A	NEG
47308	Canine	2019	1187	NEG	NEG	N/A	NEG
47309	Canine	2019	1160	NEG	NEG	N/A	NEG
47310	Canine	2019	835	NEG	NEG	N/A	NEG
47311	Canine	2019	1050	NEG	NEG	19.83	POS
47312	Canine	2019	943	NEG	NEG	37.94*	NEG
47313	Canine	2019	2899	NEG	NEG	N/A	NEG
47314	Canine	2019	2569	NEG	NEG	30.12	POS
47315	Caprine	2018	4573	NEG	NEG	N/A	NEG
47316	Canine	2019	1900	NEG	NEG	N/A	NEG
47317	Canine	2019	2861	NEG	NEG	N/A	NEG
47318	Canine	2019	587	NEG	NEG	38.13*	NEG
47319	Canine	2018	4340	POS	POS	15.19	POS
47320	Canine	2019	1876	NEG	NEG	N/A	NEG
47321	Canine	2019	1898	NEG	NEG	N/A	NEG
47322	unknown	2019	579	NEG	NEG	N/A	NEG
47325	Canine	2019	1910	POS	POS	18.35	POS
47326	Canine	2018	5947	POS	POS	26.17	POS
47327	Canine	2019	2657	POS	POS	23.07	POS
47328	Canine	2018	4755	POS	POS	19.58	POS
47329	Canine	2019	104	POS	POS	24.84	POS
47330	Kudu	2018	1710	NEG	NEG	N/A	NEG
47407	Canine	2018	5114	POS	POS	15.36	POS
47408	Canine	2019	1903	POS	POS	21.69	POS
47409	Canine	2019	2658	POS	POS	21.95	POS
47410	Kudu	2019	551	POS	POS	16.04	POS
47411	Canine	2018	4567	NEG	NEG	N/A	NEG
47412	Caprine	2019	860	NEG	NEG	N/A	NEG
47413	Canine	2019	2415	POS	POS	20.90	POS

47414	Canine	2018	4118	POS	POS		22.72	POS
47415	Canine	2019	2570	POS	POS		18.82	POS
47416	Canine	2018	4461	POS	POS		17.91	POS
47417	Caprine	2018	1168	NEG	NEG		N/A	NEG
47418	Kudu	2018	6531	NEG	NEG		N/A	NEG
47419	Canine	2018	1676	POS	POS		17.08	POS
47420	Caprine	2018	4831	POS	POS		17.69	POS
47421	Caprine	2018	1205	POS	POS		15.32	POS
47422	Canine	2019	1899	POS	NEG		N/A	NEG
47423	Canine	2019	1518	NEG	NEG		N/A	NEG
47424	Canine	2019	2860	POS	POS		20.30	POS
47425	Canine	2019	77	NEG	NEG		36.69*	NEG
47426	Kudu	2018	5516	NEG	NEG		37.34*	NEG
47536	Caprine	2019	119	POS	POS		17.25	POS
47538	Canine	2019	1104	POS	POS		21.99	POS
47539	Canine	2018	4464	POS	POS		22.03	POS
47540	Canine	2019	588	POS	POS		17.70	POS
47541	Canine	2019	88	POS	POS		21.98	POS
47542	Canine	2018	6571	POS	POS		19.25	POS
47543	Caprine	2019	2765	POS	POS		18.48	POS
47544	Canine	2019	578	POS	POS		26.31	POS
47545	Kudu	2018	6632	NEG	NEG		22.89	POS
47546	Canine	2019	1183	POS	POS		17.17	POS
47547	Canine	2019	1260	POS	POS		25.53	POS
47548	Canine	2018	102	POS	POS		21.47	POS
47549	Canine	2018	4948	POS	POS		17.42	POS
47550	Caprine	2018	4830	POS	POS		19.24	POS
47551	Caprine	2018	2771	POS	POS		18.50	POS
47552	Caprine	2019	141	POS	POS		16.15	POS
47553	Canine	2018	87	NEG	NEG		36.42*	NEG
47554	Canine	2019	2458	POS	POS		16.87	POS
47555	Caprine	2019	502	POS	POS		34.36	POS
47593	Kudu	2018	6569	NEG	NEG		N/A	NEG
47594	Canine	2019	2766	POS	POS		17.90	POS
47595	Canine	2018	4710	POS	POS		18.20	POS
47596	Canine	2019	80	POS	POS		20.20	POS
47597	Canine	2019	73	POS	NEG		N/A	NEG
47598	Kudu	2019	2041	POS	POS		17.22	POS
47599	Canine	2019	67	POS	NEG		N/A	NEG
47600	Canine	2018	71	POS	POS		20.61	POS
47601	Caprine	2019	2394	POS	POS		16.31	POS
47602	Canine	2019	61	POS	NEG		N/A	NEG
47603	Caprine	2019	139	POS	POS		17.84	POS
47604	Kudu	2019	2282	POS	POS		20.60	POS
47605	Canine	2019	1328	POS	POS		22.15	POS
47606	Canine	2019	2393	NEG	NEG		N/A	NEG
47607	Caprine	2019	4720	POS	POS		19.79	POS

47608	Caprine	2018	3660	**POS**	**POS**		17.20	**POS**
